# Can Pitch Mismatch Be Diagnosed in Children Who Use Electric-Acoustic Stimulation?

**DOI:** 10.7759/cureus.10338

**Published:** 2020-09-09

**Authors:** Vahid Moradi, Saeid Farahani, Farzaneh Fatahi, Saleh Mohebbi, Hessam Emamdjomeh

**Affiliations:** 1 Department of Audiology, School of Rehabilitation Sciences, Tehran University of Medical Sciences, Tehran, IRN; 2 Skull Base Research Center, the Five Senses Institute, Iran University of Medical Sciences, Tehran, IRN; 3 ENT and Head & Neck Research Center, Iran University of Medical Sciences, Tehran, IRN

**Keywords:** bimodal cochlear implant, pitch mismatch, sound induced flash illusion

## Abstract

Introduction

Pitch mismatch is one of the most important problems of users of bimodal cochlear implants, which affects their life satisfaction. Children with acquired hearing loss cannot explain their pitch mismatch problems, as they have had no auditory experience. This study tries to diagnose pitch mismatch in these children through the sound-induced flash illusion test.

Materials and methods

In this study, 20 children with a bimodal cochlear implant and 20 children with normal hearing, within the age range of 8 to 13 years old, were examined using the sound-induced flash illusion test. In this test, participants received one flash with one to four beep sounds, and they were asked to indicate the number of perceived flashes.

Results

The results revealed that in the bimodal implantation group, when the flash was provided with one beep, at certain frequencies, children expressed that they saw two flashes. However, the results were not the same in children with normal hearing.

Conclusion

The results indicated that at frequencies where the auditory information of the hearing aids and those of the cochlear implants overlap, pitch mismatch develops, which can significantly affect the auditory performance of bimodal users.

## Introduction

Children with severe to profound hearing loss have no experience of hearing. Thus, when they receive hearing aids or cochlear implantation, according to the sound they get from the amplifier, the auditory nerves bring the sound to the temporal cortex, where brain mapping occurs for input frequencies [1]. However, in children and adults with post-lingual hearing loss, since they have previously experienced normal hearing, they can explain the difference in hearing before and after hearing loss in terms of the quality of the sound and features such as pitch or loudness [2]. Children with a unilateral cochlear implant have many difficulties in perceiving music and speech recognition, particularly in noisy situations, as well as in localization [3-4]. Thus, it is recommended that they either use a hearing aid for one ear (bimodal stimulation) or use a cochlear implant for the second ear (bilateral cochlear implant) [5]. When a hearing aid is used in the ear opposite the ear with the cochlear implant, localization, music perception, and speech recognition improve [6]. This improvement is due to the low-frequency information provided by hearing aids and have referred to the effect of fundamental frequency [7].

Despite all the advantages of acoustic-electric stimulation, the users of acoustic-electric stimulation are not permanent users of hearing aids, as they are not fully satisfied with the aids, which is due to the pitch mismatch between the ears [8]. Studies have referred to various reasons for this problem, among which we can mention the following: residual hearing of the ear opposite to the implanted ear [9], imbalance of the loudness between the ears [10], pitch mismatch between ears [8], cochlear dead regions [11], differences in the features of processing circuit and digital delay [12], non-use of rehabilitation programs [13]. However, most bimodal implant users do not use the hearing aid permanently since they complain that, in some cases, the hearing aid worsens their audibility and they receive different pitches in the two ears. This is due to the different stimulation patterns of the auditory nerve, the stimulation site, and the different processing algorithms in the hearing aid and cochlear implant [14]. In cochlear implants, electrodes cannot be inserted deep into the apex of the cochlea, thus limiting the stimulation of the low-frequency areas at the apex of the cochlea. However, the hearing aid could transfer the sound easily to the apex of the cochlea to allow for hearing low-pitched frequencies. When using a hearing aid, the acoustic information enters the ear canal after amplification and then reaches the cochlea through the eardrum and ossicles. Eventually, the fibers of the auditory nerve are stimulated. However, in cochlear implantation, the sound is transmitted directly to the auditory nerve through electrical pulses. These factors cause a difference between the pitch of the sound received in the cochlear implant and the ear with the hearing aid [8]. According to various studies, pitch mismatch may significantly affect the auditory performance of cochlear implant users, especially in noisy environments, thus preventing bimodal implant users from using hearing aids regularly [15]. In adults and children with post-lingual hearing loss, the pitch mismatch can be reduced by fine-tuning and rehabilitation programs [12]. However, in children with prelingual hearing loss, as they have had no experience of normal hearing, they cannot accurately compare the pitch between the cochlear implant and hearing aids, and, practically, the pitch mismatch in this population has remained somewhat ambiguous [16]. Most of the studies on pitch mismatch have been performed on adults or children with post-lingual hearing loss who have had the experience of normal hearing [17-18]. However, no objective or subjective tests have been conducted on individuals to accurately determine the pitch mismatch in this group.

In 2002, Shams et al. introduced the sound-induced flash illusion (SIFI). In this phenomenon, an acoustic stimulus can affect the perception of the visual stimulus. The test provides the participant with a flash stimulus, which is accompanied by one to four beep sounds. Then, the participant is asked how many flashes they have seen [19]. For example, when one beep accompanies a flash, the participant would perceive only one flash. However, if two or three beeps accompany one flash, the participant would perceive two or three flashes, respectively. Hence, the flash illusion is induced by acoustic stimuli [19]. This illusion indicates the integration of visual and auditory information at the auditory and visual cortex [20]. Studies have indicated that the SIFI phenomenon is not affected by factors such as frequency [19], feedback training [21], and hearing aid digital delay [22]. Rather, it has been attributed to the modulation of activity at the levels of the visual cortex and the superior temporal gyrus, with neuroimaging techniques such as fMRI confirming the increased activity in the visual cortex during SIFI [19].

Accordingly, in this study, we intend to diagnose pitch mismatch in bimodal implant children through the integration of auditory and visual sensory information. Hence, one flash and one beep sound are provided for the child. According to Shams et al., in this phase the child should report only one flash. However, if the child reports two flashes, it may be assumed that the child has heard two beep sounds due to the pitch mismatch between the hearing aid and the cochlear implant. This test is performed for the frequencies of 125, 250, 500, 750, 1000, and 1500 Hz.

## Materials and methods

In this study, 20 prelingual hearing loss children with a bimodal cochlear implant (11 females, 8 males) within the age range of eight to 12 (mean of 10 and SD of 1.25) and 20 children with normal hearing (9 females, 11 males) within the age range of eight to 13 (mean of 10.03 and SD of 1.09), who had no middle ear infection in the tympanometry test [23], mental disorder [24] or visual disorders [25], were included in the study. In the normal group, the mean hearing threshold at the frequencies 125 to 8000 Hz for the right and left ears is 7.81 (SD= 3.99) and 6.35 (SD= 3.52), respectively. In the bimodal group, the cochlear implant surgery should have been performed at least three years ago, and the patient should have used a hearing aid in the opposite ear for at least six months [13].

Table 1 reports the age, gender, cause of hearing loss, the implanted ear (left/right), cochlear implant usage years, cochlear implant type, stimulation strategy, hearing aid usage years, and the mean hearing threshold at 250, 500, 1000, 2000 Hz frequencies for the opposite ear (non-implanted ear) for bimodal cochlear implant users.

**Table 1:** Demographic data for bimodal cochlear implant (CI) users F: female, M: male, yr: years, PTA: pure tone average: decibel, Ave: average, SD: standard deviation, Hz: Hertz, ACE: advanced combination encoder, SPEAK: spectral peak

2000 (Hz)	1000 (Hz)	750 (Hz)	500 (Hz)	250 (Hz)	HA Usage years	CI Strategy	CI type	CI usage years	CI Ear	etiology	Gender	Age (yr)	Subjects
100	95	95	80	70	6.70	ACE	CI24RE	6.40	Right	unknown	F	11	1
95	100	90	75	80	6.80	ACE	CI24RE	5.90	Right	Genetic	M	10	2
120	120	100	100	90	8.40	ACE	CI24RE	6.00	Right	unknown	M	10	3
120	100	90	90	85	6.90	SPEAK	CI24R	7.20	Right	unknown	F	9	4
100	80	75	80	70	10.50	SPEAK	CI24R	9.30	Right	meningitis	F	12	5
120	110	90	80	75	2.00	ACE	CI24RE	6.90	Left	Waardenburg syndrome	F	11	6
120	105	85	80	70	4.50	ACE	CI24RE	6.40	Right	unknown	M	10	7
110	95	85	75	80	5.80	ACE	CI24RE	5.10	Right	unknown	F	9	8
110	95	85	85	70	3.70	ACE	CI24RE	6.10	Right	unknown	M	8	9
120	120	90	75	75	9.30	ACE	CI24RE	6.60	Right	Mumps	M	11	10
110	105	90	80	75	1.00	ACE	CI24RE	6.40	Right	unknown	F	10	11
105	80	90	75	75	5.70	SPEAK	CI24R	7.80	Left	Genetic	F	11	12
120	105	95	85	70	7.60	SPEAK	CI24R	9.00	Left	unknown	F	12	13
120	120	105	80	55	6.90	ACE	CI24RE	5.80	Right	unknown	M	9	14
120	120	120	100	90	3.70	ACE	CI24RE	4.40	Right	Ototoxiciy	M	8	15
120	110	75	80	75	4.50	ACE	CI24RE	4.70	Right	unknown	F	8	16
120	115	95	100	100	5.00	SPEAK	CI24RE	6.00	Right	CMV	M	10	17
105	95	75	75	60	7.30	SPEAK	CI24R	7.30	Right	unknown	M	11	18
120	120	100	100	85	5.80	ACE	CI24RE	5.90	Left	Pendred’s syndrome	F	9	19
110	85	85	80	75	8.30	SPEAK	CI24R	7.70	Right	unknown	F	11	20
113.25 (8.47)	103.75 (13.26)	90.75 (10.67)	83.75 (9.16)	76.25 (10.37)	6.02 (2.36)			6.54 (1.25)				10 (1.25)	Ave (SD)

In the group of children with bimodal implantation, all children had the Cochlear Nucleus® implant (Cochlear Americas, Lone Tree, CO) so that the effect of different processors on the test would be controlled. All participants were tested in terms of the aided hearing threshold at the frequencies of 250 to 4000 Hz for electrical stimulation alone, or hearing aid alone, so that the amplified hearing threshold at the normal level would be confirmed. Table 2 presents the mean aided hearing threshold. In all children with bimodal implantation, the loudness was balanced between the two ears according to the National Acoustic Laboratories (NAL) approach for the frequencies of 125 to 1500 Hz [26].

**Table 2:** Aided hearing threshold for cochlear implants and hearing aids Frq: frequency, dB: decibel, Hz: hertz

Aided hearing threshold with cochlear implant (dB)					Aided hearing threshold with hearing aid (dB)					
4000 (Hz)	2000 (Hz)	1000 (Hz)	500 (Hz)	250 (Hz)	4000 (Hz)	2000 (Hz)	1000 (Hz)	500 (Hz)	250 (Hz)	Frq
30	30	35	30	30	85	50	30	30	25	1
35	35	30	40	30	80	55	30	25	30	2
30	35	30	30	30	NR	80	40	60	40	3
30	35	40	40	30	70	45	30	40	30	4
30	30	30	35	35	85	30	35	35	30	5
30	40	45	40	35	NR	40	25	25	35	6
30	30	35	35	30	NR	75	35	40	30	7
35	35	40	35	30	85	50	30	40	30	8
25	30	35	35	30	NR	65	30	45	30	9
30	30	40	40	35	NR	65	40	60	45	10
30	35	35	40	30	80	55	35	40	30	11
30	30	30	35	35	90	45	30	45	30	12
35	35	40	35	35	85	50	30	30	35	13
35	30	40	45	30	NR	75	45	60	30	14
30	25	35	40	30	NR	80	55	55	40	15
30	40	35	35	30	80	45	35	40	35	16
30	30	35	35	40	85	55	45	60	40	17
35	40	40	40	30	90	60	25	30	25	18
35	30	30	35	30	NR	80	60	75	40	19
35	35	45	40	30	75	45	40	40	25	20

**Figure 1:**
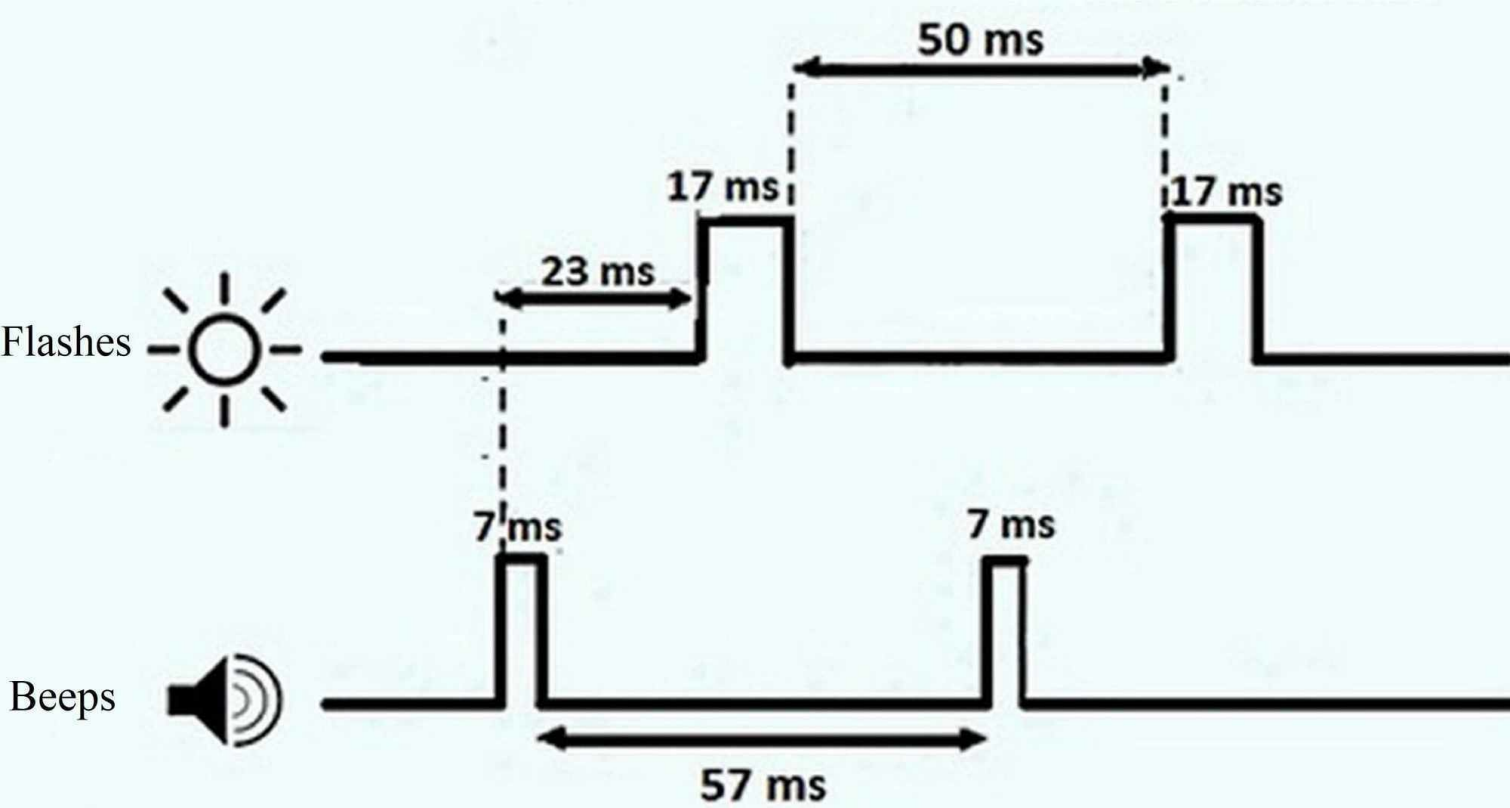
Temporal profile of the flash(s) and beep(s) stimuli in the SIFI test SIFI: sound-induced flash illusion

In order to ensure the visual health of the participants, all subjects were assessed using a Snellen visual chart. Also, the tympanometry test was performed to rule out middle ear infection in all participants. Written informed consent was obtained from the participants. The study was approved by the Human Research Ethics Committee of the University of Medical Sciences (IR.TUMS.FNM.REC.1398.139). For the hearing stimulus (beep), the frequencies of 125, 250, 500, 750, 1000, and 1500 Hz were played with a duration of 7 ms at 95 dB SPL by a speaker in the center of the monitor. The time interval between the beeps was 57 ms, and the beeps were always played 23 ms before the flash. The flash stimulation was played with a duration of 17 ms and the luminance of 108 cd/m^2^ at the angle of 2° out of 5° of the visual field eccentricity on the black display of a laptop with a luminance of 0.02 cd/m^2^. The time interval between the flashes was 50 ms [19]. Details are shown in Figure 1.

To perform the test, participants sat on a chair 58 cm away from the laptop display (model ASUS X550). They were asked to fixate their eyes on the display continuously and to report the number of flashes they could see. The test was conducted in a dimly lit room and in a quiet environment, where the participants were asked to turn off their cellphone in the room. In the test, a flash was played along with zero to four beep sounds presented via the speaker at each frequency (125, 250, 500, 750, 1000, and 1500 Hz), where the participants were asked to indicate the number of flashes they could observe. Each test was performed randomly five times per participant for each frequency. Then, the results of both groups were recorded at different frequencies and then analyzed. All analyses were performed by SPSS version 22 (IBM Corp, Armonk, NY). Analysis of variance (ANOVA) was used to compare the mean number of flashes perceived by each of the two groups. The Tukey honest significance test (HSD) post-hoc was used for pairwise comparison when ANOVA was significant. The significance level was considered 0.05.

## Results

**Figure 2:**
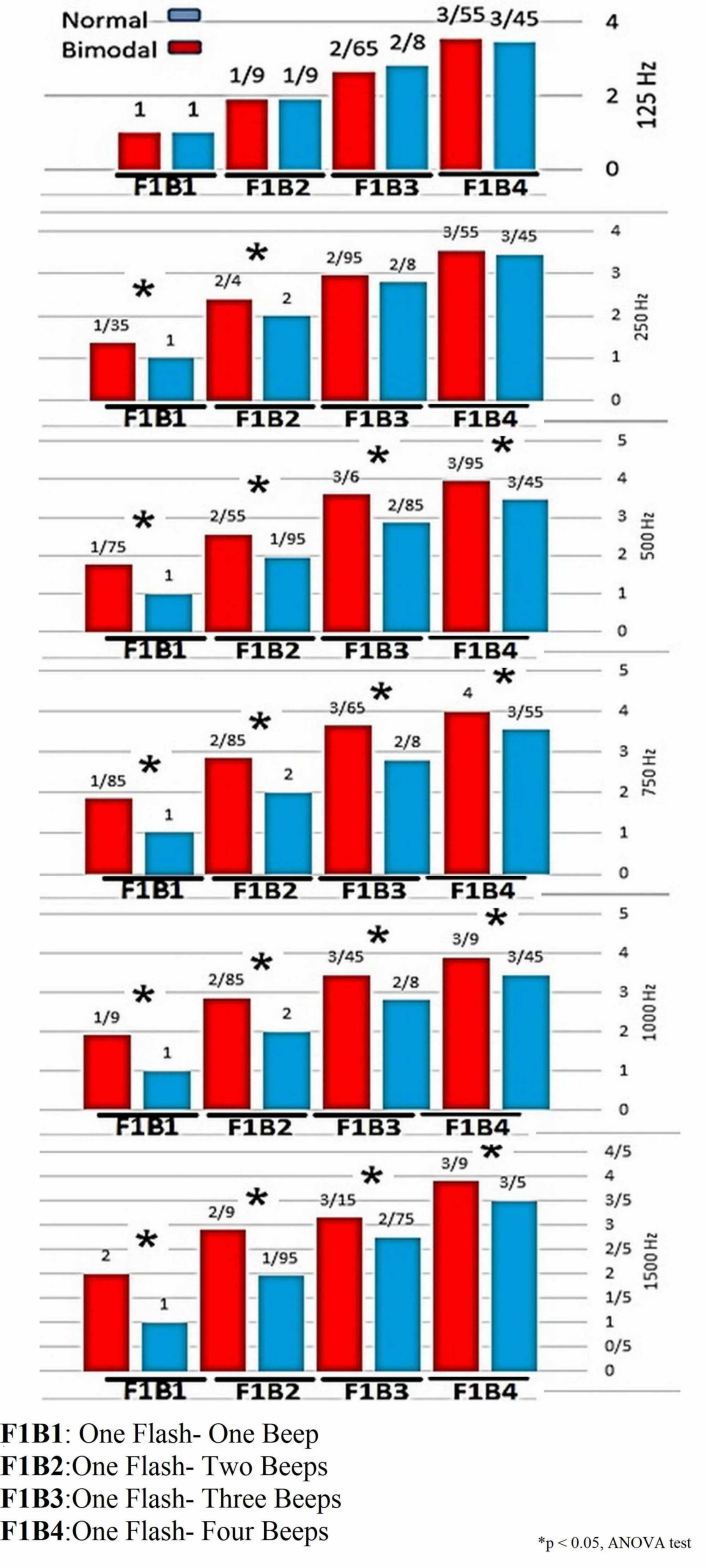
The mean of the perceived number of flashes for different frequencies in the bimodal cochlear implant group (Red) and the normal hearing group (Blue) In all frequencies for the F1B0 trial, the numbers of perceived flashes are very close to each other, but when the number of beeps increases, the results for the different frequencies are highly variable. At the frequency of 125 Hz for all trials, there is no significant difference between the two groups. At the frequency of 250 Hz, a significant difference between the groups can be observed only for the F1B1 and F1B2 trials. From the frequency of 500 Hz to 1500 Hz, there was a significant difference in the number of perceived flashes for all trials. As can be seen in the F1B1 and F1B2 trials, with the increase in frequency, the mean difference in the number of perceived flashes between the participants increased.

There is no significant difference between the aided hearing thresholds between ears in the bimodal group. The results of normal participants and participants with a bimodal cochlear implant revealed that at different frequencies and various trials, there is a significant difference between the two groups. Figure 2 provides the mean of the perceived number of flashes for different frequencies at the Y-axis.

As can be seen at all frequencies for the F1B0 trial, the numbers of perceived flashes are very close to each other, but when the number of beeps increases, the results for the different frequencies are highly variable. At the frequency of 125 Hz for all trials, there is no significant difference (p-value > 0.05) between the two groups. On the other hand, at the frequency of 250 Hz, a significant difference (p-value < 0.05) between the groups can be observed only for the F1B1 and F1B2 trials. From the frequency of 500 Hz to 1500 Hz, there was a significant difference (p-value < 0.05) in the number of perceived flashes for the F1B1, F1B2, F1B3, and F1B4 trials. As can be seen in the F1B1 and F1B2 trials, with the increase in frequency, the mean difference in the number of perceived flashes between the participants with normal hearing and bimodal implant users increased. However, for the F1B3 and F1B4 trials, no specific pattern could be seen upon frequency elevation and the mean number of perceived flashes is very variable in both groups, which is due to applying a more difficult test for these two trials.

## Discussion

The present results show that pitch mismatch increases with increasing frequency. This mismatch is due to differences in cochlear implants and hearing aids. For most cochlear implant users, due to the impossibility of inserting the electrode deep into the apex of the cochlea, and the sound processing algorithms, pitch perception for frequencies below 300 Hz is limited, so cochlear implants cannot use frequencies below 300 Hz. This limitation causes them to lose many temporal fine structures related to F0 [8]. In the SIFI test, bimodal users' responses for all frequencies are more variable than the control groups'. Since users of bimodal cochlear implants are largely deprived of frequency data below 300 Hz, at the frequency of 125 Hz, only the hearing aid can transmit the data from this frequency range to the auditory cortex. Thus, there was no significant difference between the bimodal cochlear implant users and the normal hearing group during the test because the bimodal users received the sound only through hearing aids and did not experience pitch mismatch. Accordingly, when one flash and one beep sound were presented for an individual, since both groups heard one sound, they reported one flash. However, at the frequencies of 250 Hz and above, the results varied between the groups, with these variations growing with the rise in frequency. At the frequencies of 250 Hz and above, the ear received stimulation from both the cochlear implant and the hearing aid, as confirmed in Figure 2. Since the sounds received by the hearing aid and cochlear implants have different pitches, bimodal participants in the F1B1 trial reported that they perceived two flashes while only one flash had been shown. However, in the normal hearing group, all participants reported only one flash. Thus, this test confirms that in the bimodal implant group, within the frequency range where the cochlear implants and hearing aids overlap, they are not completely matched, causing the bimodal implant group to receive two different pitches for a single tone [17]. Then, as the frequency of acoustic stimuli increased, the hearing aid could not cover the hearing loss severity, as confirmed in Table 2 through the results of aided hearing threshold using the hearing aid. It can be inferred that in the cochlear implant ear, at all frequencies, the hearing threshold fell within the normal range of the speech banana spectrum in the aided mode. however, the aided hearing threshold by the hearing aid exceeded the normal range upon the rise in the frequency. Thus, the patient with hearing loss could not receive the speech sounds provided by the hearing aid completely. This difference in the amplified range increased with frequency elevation [18]. For example, for participant No. 19, all frequencies in the cochlear implant ear were heard at the intensity level of 30 to 35 dB, but in the hearing aid ear, the amplified hearing threshold was only normal at the frequency of 250 Hz, as the frequency increased, the amplified hearing threshold was aggravated significantly. As observed in Table 2, at the frequency of 250 Hz, the mean difference between the number of flashes perceived between the two groups was 0.35 while at the frequencies of 500, 750, 1000, and 1500 Hz, the difference was 0.75, 0.85, 0.90, and 1.00, respectively. Thus, as the frequency increased, the mean difference between the number of perceived flashes between the two groups rose sharply, which is due to the inadequate coverage of the hearing aids at higher frequencies, resulting in maximum loudness and pitch mismatch between the ears [17].

According to the mean number of perceived flashes between the two groups, we found that in the F1B3 and F1B4 modes, there was a significant difference between the bimodal group and the normal subjects. In this regard, studies have shown that the F1B3 and F1B4 trials are difficult even for people with normal hearing [19]. However, in bimodal patients, due to the loudness and pitch mismatch between the ears, the scores recorded at frequencies of 250 Hz and above are more variable. It can be concluded that in electric-acoustic stimulation at frequencies where the cochlear implant and the hearing aid interact since the loudness and pitch cannot be matched between the two ears completely, it makes bimodal implant users experience pitch mismatch, which affects the binaural integration of auditory cues [27] and prevents patients from using the hearing aid permanently [18]. All these cause them to prefer hearing only through a unilateral cochlear implant. Also, it was observed that in the SIFI test, there was a significant difference between the results of children with normal hearing and bimodal implantation. Thus, this test can be used to diagnose pitch mismatch between the ears. Nevertheless, this test helps us identify the pitch mismatch at various frequencies in order to reduce the problems of pitch mismatch in bimodal users by fine-tuning the loudness balance while also using rehabilitation programs. In case the pitch mismatch persists, it is recommended to eliminate the frequencies that cause mismatch by reducing the gain of the hearing aid, so that bimodal users receive frequencies only through the cochlear implant.

## Conclusions

In conclusion, the findings of the study indicated that in bimodal cochlear implant users, loudness and pitch cannot be matched between the two ears completely. The SIFI test helps specialists manage pitch mismatch at various frequencies. Therefore, conducting the SIFI test in cochlear implant centers is recommended.
